# Evaluation of Negative Pressure Dressings for Closed Surgical Incisions in Decreasing Surgical Site Infections After Emergency Laparotomy: A Randomized Controlled Study

**DOI:** 10.7759/cureus.67500

**Published:** 2024-08-22

**Authors:** Kartik Sahni, Shridhar Hosamani, Deepak Ghuliani, Shikha Baisoya

**Affiliations:** 1 Department of Gastrointestinal Surgery, All India Institute of Medical Sciences, New Delhi, IND; 2 Department of Surgery, Maulana Azad Medical College, New Delhi, IND

**Keywords:** wound dehiscence, conventional wound dressings, emergency laparotomy, surgical site infection, negative pressure dressing

## Abstract

Objectives

The aim of this study is to compare the effectiveness of negative pressure dressings (NPDs) versus conventional dressings for closed surgical incisions after emergency midline laparotomy, focusing on their impact on surgical site infection (SSI) rates, wound dehiscence, hospital stay duration, and cosmetic outcomes.

Methods

The randomized controlled study was conducted over 24 months, involving 80 patients aged 18-65 years who had peritonitis and underwent emergency midline laparotomies. Patients with diabetes mellitus, a BMI >35 kg/m², immunocompromised conditions, or those requiring re-exploration within 30 days of surgery were excluded. The participants were randomly assigned into two groups using a computer-generated randomization table: Group A, the case group, consisted of 40 patients who received NPDs, while Group B, the control group, included 40 patients who received conventional dressings. Data were recorded in Microsoft Excel (Microsoft Corporation, Redmond, WA, USA) and analyzed using IBM SPSS Statistics for Windows, Version 25.0 (Released 2017; IBM Corp., Armonk, NY, USA), with a p-value of <0.05 considered statistically significant.

Results

The overall occurrence of SSIs within the 30-day follow-up period was significantly lower in the NPD group compared to the conventional dressing group (30% vs. 70%, p < 0.05). The mean duration of hospital stay was 14.85 ± 10.43 days for the NPD group and 15.4 ± 9.75 days for the control group, with no statistically significant difference (p = 0.712). The mean Vancouver Scar Scale score was 5.3 ± 2.47 in the NPD group and 6.5 ± 2.14 in the control group, also showing no statistically significant difference (p = 0.11).

Conclusions

NPDs significantly reduced the incidence of SSIs compared to conventional dressings, but they did not have a significant impact on scar cosmesis or the duration of hospital stay.

## Introduction

Surgical site infection (SSI) is defined as a wound infection that occurs within 30 days of surgery, including superficial or deep incisional SSIs and organ/space SSIs, or within one year if an implant is used [1]. SSIs have been a concern in surgical practice since prehistoric times [2]. Although the incidence of SSIs has decreased with the advent of antibiotics, they still cause significant morbidity and impose substantial costs and resource burdens on both patients and healthcare providers. SSIs result in delayed wound healing, prolonged hospital stays, late return to normal activities and work, poor scar formation due to healing by secondary intention, increased discomfort, and reduced quality of life. The incidence of SSIs following open abdominal surgeries ranges from 10% to 35%, influenced by factors such as the degree of contamination during surgery, age, immunocompromised state, duration of surgery, diabetes mellitus, and use of immunosuppressive therapy, etc. [3,4].

To prevent SSIs, various methods have been employed, including saline and gauze dressings, antibiotic prophylaxis, suction drain placement, wound protection devices, antibiotic-coated sutures, incisional wound irrigation, and advanced dressings [5]. Despite significant progress in the medical sciences over nearly two centuries, SSI prevention remains a major concern and continues to be a leading cause of nosocomial morbidity and mortality.

Negative pressure dressings (NPDs), initially used for open abdominal wounds, have more recently been applied to closed surgical incisions [6]. The vacuum-assisted closure (VAC) device is commonly used for this purpose. NPDs operate on the principle that negative pressure helps remove interstitial fluid, decrease localized edema, and increase blood flow, thereby reducing tissue bacterial levels and inducing mechanical cell deformation. This process promotes protein and matrix molecule synthesis, which enhances cell proliferation [7-9]. When applied to closed surgical incisions, NPDs serve several functions: holding the incision edges together, redistributing lateral tension, reducing edema, protecting the surgical site from external infections, optimizing blood flow, and aspirating accumulated fluid. These actions collectively facilitate bacterial clearance and support the healing process [6-8].

Several studies have evaluated the impact of NPDs on abdominal wounds, showing a reduction in postoperative SSI incidence. However, the application of NPDs to closed surgical wounds requires further investigation. This randomized controlled study aims to compare the effectiveness of NPDs versus conventional dressings for closed surgical incisions after emergency midline laparotomy in terms of SSI rates, wound dehiscence, hospital stay duration, and cosmetic outcomes [10,11].

## Materials and methods

This randomized controlled study was conducted at the Department of Surgery, Maulana Azad Medical College, New Delhi, India, and the associated Lok Nayak Hospital over a 24-month period from April 2018 to April 2020. The study included patients aged 18-65 years undergoing emergency midline laparotomies for peritonitis. Exclusion criteria included diabetes mellitus, any immunocompromised state, a BMI >35 kg/m², or the need for re-exploration within 30 days of surgery. Emergency laparotomy decisions were based on a thorough history, physical examination, routine blood investigations, chest X-ray, abdominal X-ray, and ultrasound of the abdomen if necessary.

All patients received antibiotic prophylaxis at the time of anesthesia induction, consisting of a single dose of co-amoxicillin-clavulanic acid (1.2 g intravenously) and metronidazole (500 mg intravenously). For procedures lasting more than four hours, a second dose of antibiotics was administered intraoperatively. The abdomen was prepped using chlorhexidine, followed by alcohol, and then painted with povidone-iodine. A midline incision was made for an emergency laparotomy, with meticulous intraoperative documentation of findings and appropriate procedures carried out based on pathology. Thorough peritoneal lavage was performed, and abdominal drains were placed. Surgeons gloves were changed before closing the abdominal wound with a fully sterile technique, and the rectus sheath was closed with loop polypropylene no. 1 in a continuous fashion in a single layer.

Patients were randomized using a computer-generated random number table and allocated by a sealed envelope method opened just before skin suturing. They were divided into two groups: 40 patients in the NPD (case) group and 40 in the conventional dressing (control) group.

**Figure 1:**
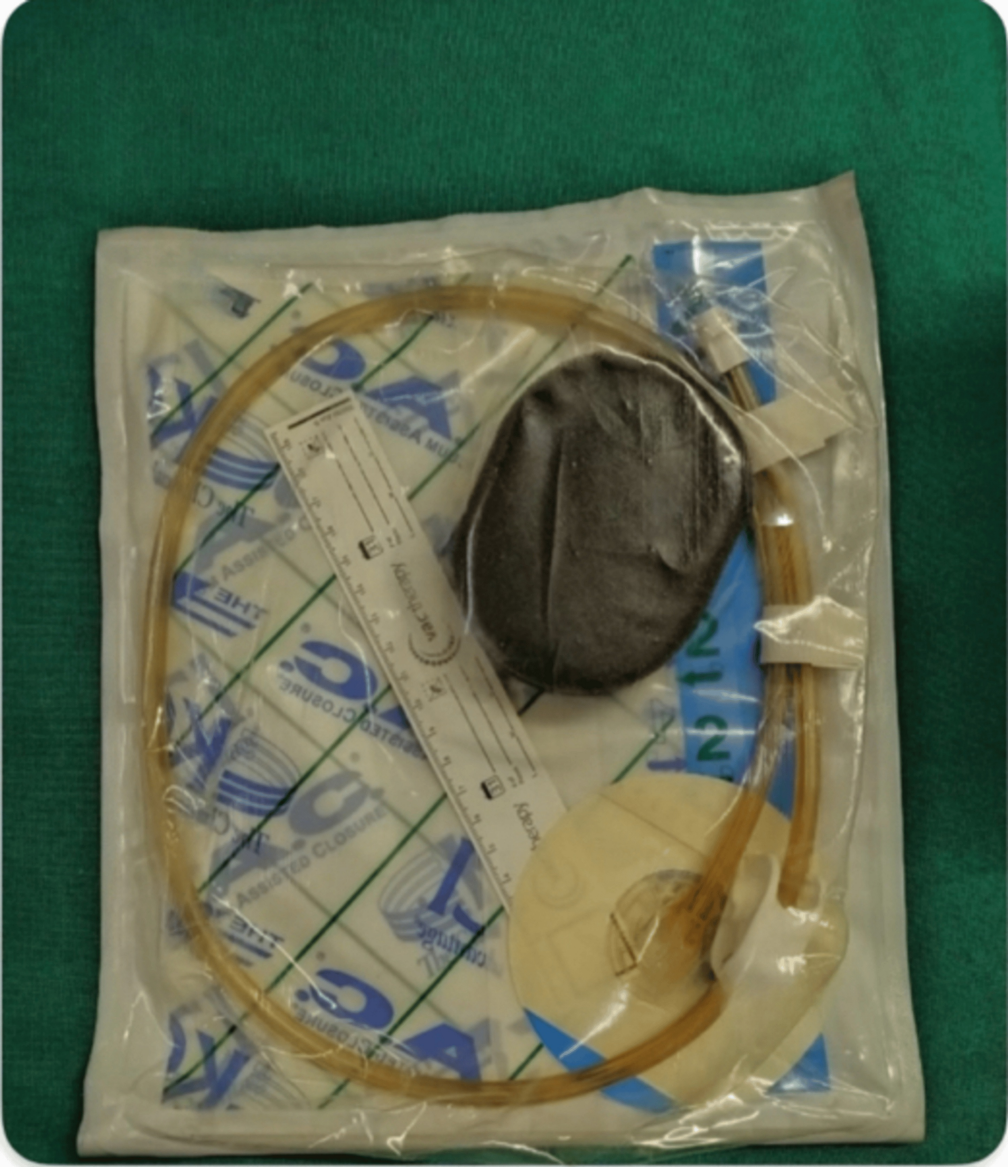
Porous foam with tubing used in VAC dressing VAC, vacuum-assisted closure

**Figure 2:**
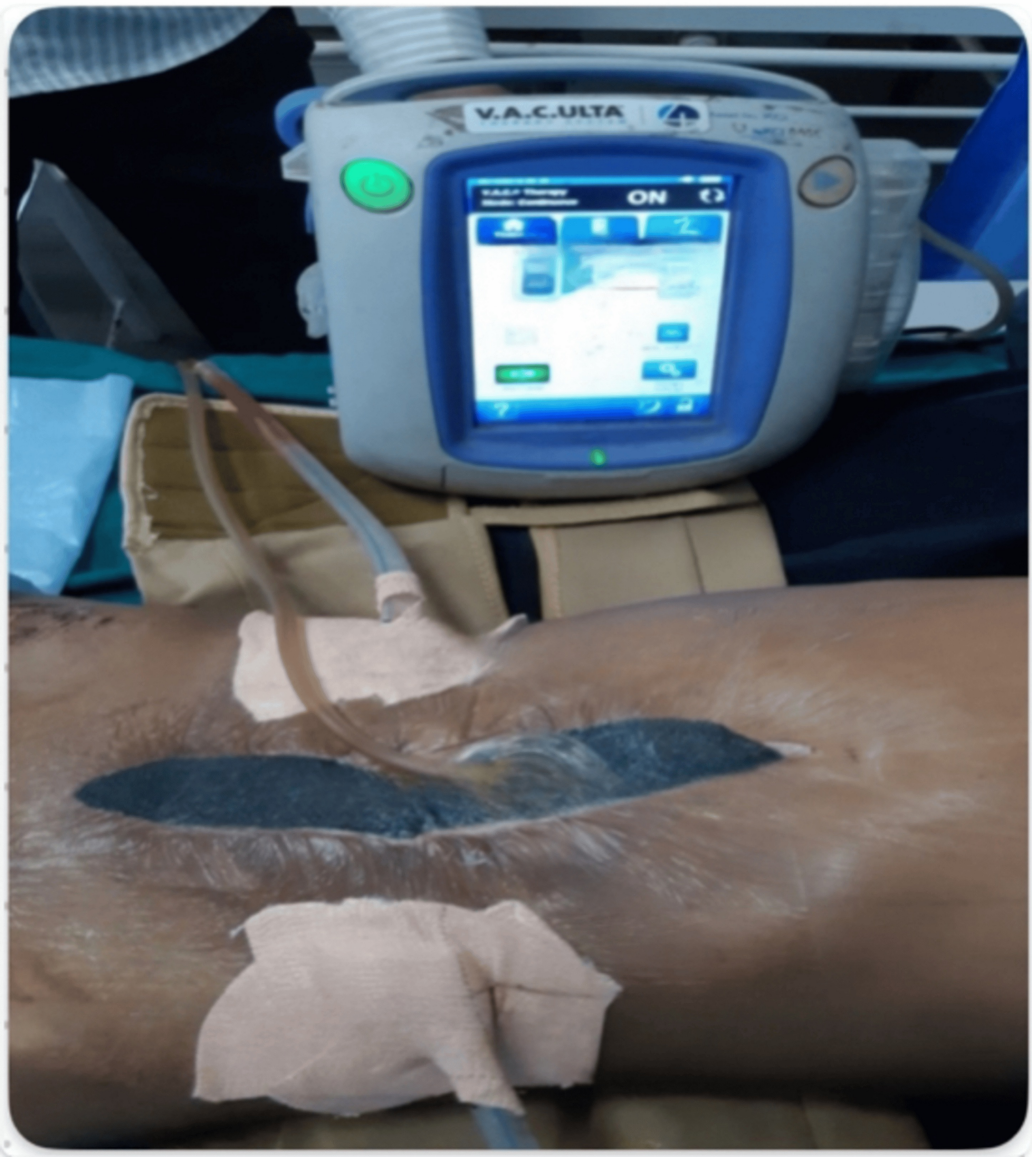
Air-tight dressing applied over the foam and connected to a VAC machine on a patient VAC, vacuum-assisted closure

**Figure 3:**
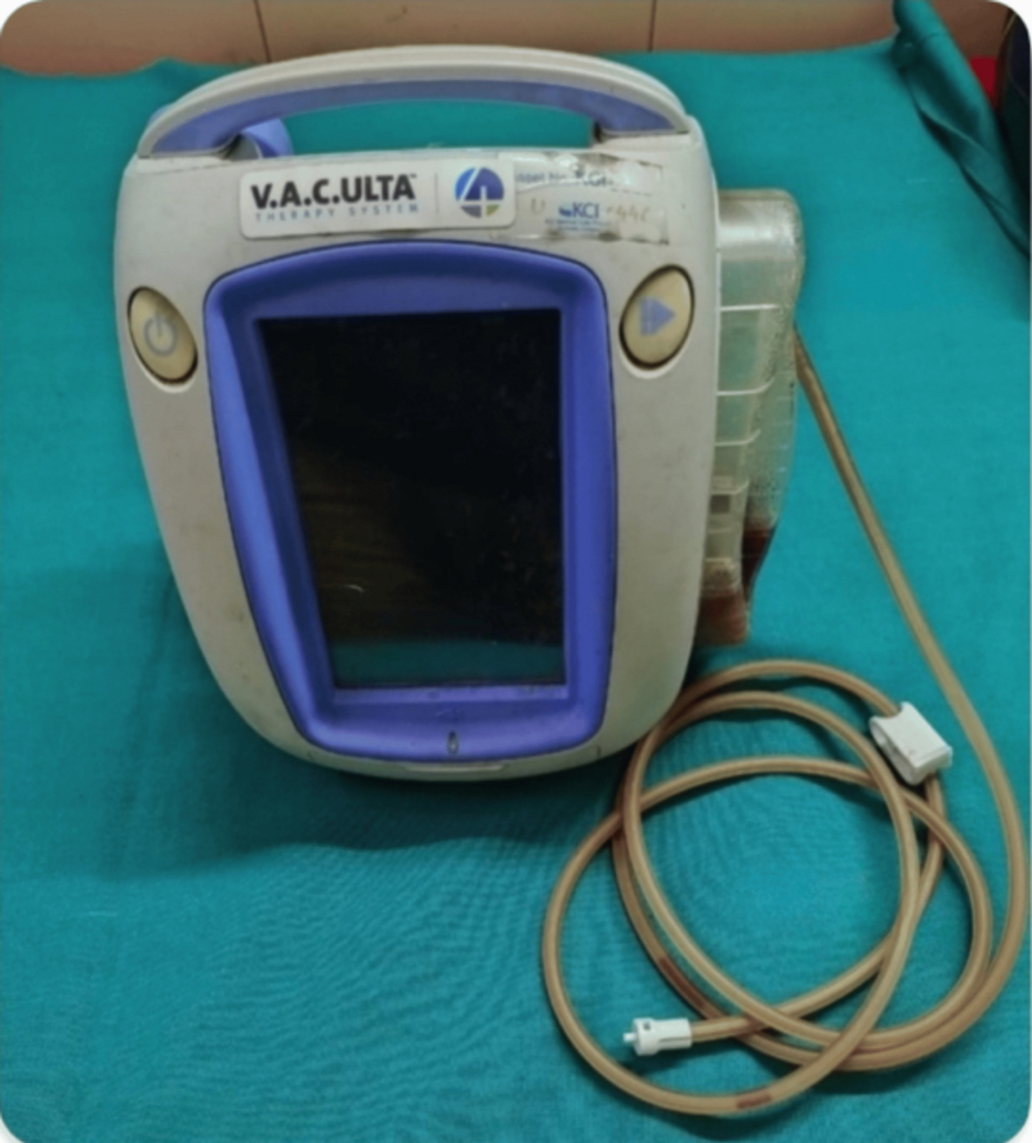
VAC machine with cannister attached VAC, vacuum-assisted closure

In the case group, NPDs were applied by inserting porous foam (Figure 1) over paraffin gauze inside the wound after leaving one to two sutures open above and below the umbilicus. An airtight dressing was applied over the foam (Figure 2) and connected to a VAC machine with a canister (Figure 3) to collect the drained contents. A continuous negative pressure of -75 mmHg was used, with dressings removed after four days and replaced with conventional dressings. If any evidence of SSI was present before four days, the NPD was removed, and collections were drained after opening the skin sutures. In the control group, conventional dressings were applied after suturing, with dressings removed after 48 hours.

Post-removal, patients in both groups were assessed for SSI (superficial or deep) as per CDC guidelines [1]. Follow-up was scheduled on postoperative days (POD) 4, 7, 14, and 30. Patients without SSI who were orally accepting and moving their bowels were discharged and followed up as scheduled. Those with SSI received appropriate management. Cosmetic outcomes were assessed at POD 30 using the Vancouver Scar Scale (VSS). If patients had open wounds on POD 30, cosmetic outcomes were reassessed on the 60th or 90th day if the wound had not healed.

Data were recorded in Microsoft Excel (Microsoft Corporation, Redmond, WA, USA) and analyzed using IBM SPSS Statistics for Windows, Version 25.0 (Released 2017; IBM Corp., Armonk, NY, USA). A p-value of <0.05 was considered statistically significant. Categorical variables were presented as numbers and percentages, while continuous variables were presented as mean ± SD and median. Normality was tested using the Kolmogorov-Smirnov test. Non-parametric tests were used if normality was rejected. For qualitative data, the Chi-square test/Fisher’s exact test was used to compare proportions. For quantitative data, the Student’s t-test/Mann-Whitney test was employed based on data distribution, with results expressed as mean and standard deviation for paired observations.

## Results

The comparison of demographic and clinical characteristics is shown in Table 1. The mean age of the patients was comparable between the case and control groups (26.75 vs. 33.8 years). The gender distribution was also similar, with 12 (30%) females and 28 (70%) males in the case group compared to 10 (25%) females and 30 (75%) males in the control group. Additionally, the duration of symptoms was comparable between the two groups, with a mean of 4.3 days in the case group and 3.6 days in the control group.

**Table 1:** Comparison of demographic and clinical characteristics between cases and controls

Parameters	Case (n = 40)	Control (n = 40)	Total	p-value	Test performed	Test value
Age (years)						
Mean ± SD	26.75 ± 8.12	33.8 ± 13.84	30.28 ± 11.76	0.15	Mann-Whitney test	147
Median (IQR)	24.5 (20-33.5)	32 (20.75-45)	26 (20-38)	-	-	-
Range	18-45	18-60	18-60	-	-	-
Gender						
Female	12 (30%)	10 (25%)	22 (27.50%)	0.723	Chi-square test	0.125
Male	28 (70%)	30 (75%)	58 (72.50%)	-	-	-
Total	40 (100%)	40 (100%)	80 (100%)	-	-	-
Duration of symptoms (days)						
Mean ± SD	4.32 ± 3.05	3.6 ± 2.16	3.96 ± 2.64	0.494	Mann-Whitney test	175
Median (IQR)	4 (2-5.25)	3 (2-5)	3 (2-5)	-	-	-
Range	0.12-12	1-8	0.12-12	-	-	-
Site of perforation/abscess						
Prepyloric perforation peritonitis	2 (10%)	1 (5%)	6 (7.50%)	-	-	-
Duodenal perforation peritonitis	2 (5%)	2 (5%)	4 (5%)	-	-	-
Jejunal perforation peritonitis	4 (10%)	0 (0%)	4 (5%)	-	-	-
Ileal perforation peritonitis	12 (30%)	16 (40%)	28 (35%)	-	-	-
Appendicular perforation peritonitis	4 (10%)	12 (30%)	16 (20%)	-	-	-
Colonic perforation peritonitis	4 (10%)	2 (5%)	6 (7.50%)	-	-	-
Gallbladder perforation peritonitis	2 (5%)	0 (0%)	2 (2.50%)	-	-	-
Ruptured liver abscess	4 (10%)	4 (10%)	8 (10%)	-	-	-
Traumatic perforation peritonitis	4 (10%)	0 (0%)	4 (5%)	-	-	-
Abdominal wall abscess with intraperitoneal extension	0 (0%)	2 (5%)	2 (2.50%)	0.534	Chi-square test	8.978
Total	40 (100%)	40 (100%)	80 (100%)	-	-	-

Table 2 presents the comparison of risk factors for SSI between the case and control groups. Both groups were evenly matched regarding these risk factors, including smoking, BMI, intraoperative contamination, preoperative hemoglobin levels, total leukocyte counts, and serum protein levels.

**Table 2:** Comparison of risk factors between cases and controls

Parameters	Case (n = 40)	Control (n = 40)	Total	p-value	Test performed	Test value
Smoking						
No	20 (50%)	30 (75%)	50 (62.50%)	0.102	Chi-square test	2.667
Yes	20 (50%)	10 (25%)	35 (37.50%)	-	-	-
BMI (kg/m²)						
Mean ± SD	22.54 ± 3.22	22.16 ± 4.01	22.35 ± 3.6	0.746	t-test	0.326
Median (IQR)	22.8 (20.375-24.1)	21.1 (19.4-23)	21.7 (20-23.725)	-	-	-
Range	15.5-31.1	17.7-33.3	15.5-33.3	-	-	-
Intraoperative contamination						
Mean ± SD	1,320 ± 1,200.26	727.5 ± 654.84	1,023.75 ± 1,000.38	0.124	Mann-Whitney test	143.5
Median (IQR)	1,100 (350-2,000)	500 (300-1,000)	700 (300-1,500)	-	-	-
Range	100-5,000	100-3,000	100-5,000	-	-	-
Hemoglobin (g/dL)						
Mean ± SD	12.07 ± 1.99	12.04 ± 1.72	12.06 ± 1.83	0.959	t-test	0.051
Median (IQR)	11.65 (10.975-12.825)	11.75 (10.725-13.25)	11.65 (10.875-13.2)	-	-	-
Range	9.1-17.5	9.3-15.6	9.1-17.5	-	-	-
Total leukocyte count (cells/mm³)						
Mean ± SD	11,956.5 ± 5,776.86	13,643.5 ± 6,297.56	12,800 ± 6,025.71	0.382	t-test	0.883
Median (IQR)	12,100 (7,937.5-14,020)	14,000(9,600-17,475)	12,800 (8,775-16,250)	-	-	-
Range	2,900-26,000	2,800-29,300	2,800-29,300	-	-	-
Serum protein(gm/dL)						
Mean ± SD	5.38 ± 0.82	5.35 ± 1.02	5.36 ± 0.92	0.892	t-test	0.136
Median (IQR)	5.2 (4.875-5.875)	5.2 (4.65-6.125)	5.2 (4.775-6.1)	-	-	-
Range	4.2-7.1	3.8-7.1	3.8-7.1	-	-	-

Table 3 compares the incidence of SSI between the case group and the control group on PODs four, seven, 14, and 30. On POD 7, four (11.76%) patients in the case group and 14 (46.67%) patients in the control group developed SSI, which was significantly higher in the control group compared to the case group (46.67% vs. 11.76%, p = 0.049). The overall incidence of SSI within 30 days was also significantly higher in the control group than in the case group (70% vs. 30%; p = 0.011).

**Table 3:** Comparison of SSI between cases and controls POD, postoperative day; SSI, surgical site infection

SSI	Case (n = 40)	Control (n = 40)	Total	p-value	Test performed	Test value
On POD 4						
Absent	34 (85%)	30 (75%)	64 (80%)	0.695	Fisher’s exact test	-
Present	6 (15%)	10 (25%)	16 (20%)	-	-	-
Total	40 (100%)	40 (100%)	80 (100%)	-	-	-
On POD 7						
Absent	30 (88.24%)	16 (53.33%)	46 (71.88%)	0.049	Fisher’s exact test	-
Present	4 (11.76%)	14 (46.67%)	18 (28.13%)	-	-	-
Total	34 (100%)	30 (100%)	64 (100%)	-	-	-
On POD 14						
Absent	28 (93.33%)	12 (75%)	40 (86.96%)	0.269	Fisher’s exact test	-
Present	2 (6.67%)	4 (25%)	6 (13.04%)	-	-	-
Total	30 (100%)	16 (100%)	46 (100%)	-	-	-
On POD 30						
Absent	28 (100%)	12 (100%)	40 (100%)	No p-value	-	-
Present	0 (0%)	0 (0%)	0 (0%)	-	-	-
Total	14 (100%)	6 (100%)	20 (100%)	-	-	-
Overall incidence within 30 days						
Absent	28 (70%)	12 (30%)	40 (50%)	0.011	Chi-square test	6.4
Present	12 (30%)	28 (70%)	40 (50%)	-	-	-
Total	40 (100%)	40 (100%)	80 (100%)	-	-	-

However, no significant difference was observed in terms of hospital stay, rectus dehiscence, and VSS assessment between cases and control groups, as shown in Table 4.

**Table 4:** Comparison of other outcomes between cases and controls VSS, Vancouver Scar Scale

Outcomes	Case (n = 40)	Control (n = 40)	Total	p-value	Test performed	Test value
Hospital stay (days)						
Mean ± SD	14.85 ± 10.43	15.4 ± 9.75	15.12 ± 9.97	0.712	Mann-Whitney test	186.5
Median (IQR)	9.5 (8-19.25)	10 (8.75-24)	10 (8-23)	-	-	-
Range	7-39	4-36	4-39	-	-	-
Rectus dehiscence						
No	32 (80%)	26 (65%)	58 (72.50%)	0.48	Fisher’s exact test	-
Yes	8 (20%)	14 (35%)	22 (27.50%)	-	-	-
Total	40 (100%)	40 (100%)	80 (100%)	-	-	-
VSS						
Mean ± SD	5.3 ± 2.47	6.5 ± 2.14	5.9 ± 2.36	0.11	Mann-Whitney test	142
Median (IQR)	4(3-7)	7(4.75-8)	6.5(3.75-8)	-	-	-
Range	3-10	3-10	3-10	-	-	-

## Discussion

In this single-center, randomized controlled trial focused on patients presenting with peritonitis, we found that negative pressure wound therapy (NPWT) for closed surgical incisions significantly reduces the incidence of SSI after emergency midline laparotomy, without affecting the duration of hospital stay, incidence of rectus dehiscence, or VSS outcomes.

NPWT has long been used in the treatment of infections like diabetic foot, necrotizing fasciitis, sternal infections after cardiac surgery, management of complex perineal wounds, and securing skin grafts. NPWT has also been utilized for treating SSI following open abdominal surgery, burst abdomen, and abdominal wall dehiscence. It is proposed that NPWT promotes wound healing by reducing lateral tension on wound edges, removing excess fluid and inflammatory mediators from the incision site, and preventing contamination by maintaining a sterile environment [6]. It promotes tissue healing by creating a hypoxic environment that causes upregulation of inflammatory cytokines, like IL-8 and IL-10, and growth factor expression, including hypoxic inducible factor-1). This local and systemic upregulation stimulates angiogenesis, and extracellular matrix deposition, and enhances granulation along with increased vessel destabilization and maturation [7-9]. Owing to favorable results for the management of SSIs, NPWT has emerged as a potential intervention to prevent SSIs following open abdominal surgery. However, there is a paucity of data on the prophylactic use of NPDs in closed laparotomy wounds. Thus, this randomized controlled study was undertaken to estimate the incidence of postoperative SSI with prophylactic NPD on closed surgical incisions in comparison to a conventional wound dressing.

The demographic distribution of the cases and controls in our study was comparable. Among other studies, O'Leary et al. [10] reported the mean age of the case group and control group as 58 years and 63 years respectively with male preponderance, as seen in our study. Another study by Murphy et al. [11] reported a mean age of 64 years in both cases and controls with male preponderance. Our study also showed male predominance as shown by other studies; however, the mean age of our study was less as compared to other studies because of the inclusion of predominantly benign conditions in our study as compared to some other studies which have included malignant conditions as well, common in older age groups.

The mean duration of symptoms in the case and control group (4.32 vs. 3.6 days) and the site of perforation/abscess were also comparable in our study. In a prospective study done by Jain et al. [12], it was found that the incidence of SSI was 46.8% (n = 90, total no. of patients = 192) of patients, followed by wound dehiscence (31.3%; n = 50) due to the late presentation of patients. In this study they found ileal perforation to be the most common site of perforation, and the same has been found in our study.

A study by Singh and Yadalwar [13] reported the incidence of SSI was higher in ileal perforation patients (54.1%). A similar study by Shen et al. [14] included patients with varied pathologies like gastrointestinal pathology (22% in the case group and 21% in the control group), pancreatic pathology (27.3% in the case group and 27.8% in the control group) and peritoneal surface malignancies (50.8% in case group and 51.1% in control group). They also divided the gastrointestinal pathologies into subgroups like esophageal, gastric, small bowel, and colorectal pathology. Similar etiologies between cases and controls are also shown in these studies, and no discernible difference was seen in the distributions of anatomical sites of disease between the two groups, as was the case in our study.

The baseline demographic and clinical characteristics were similar between case and control groups, ensuring that the results were attributable to specific interventions rather than chance [15]. Risk factors for SSIs, such as smoking, BMI, hemoglobin levels, and intraoperative contamination, did not differ significantly between groups, avoiding confounding factors affecting wound healing. Similar results were also seen in several previous studies, studying the effect of NPD [10,11,14,16].

The regression analysis to determine the factors associated with SSI showed that only serum protein was a significant risk factor for SSI (total mean ± SD: 5.36 ± 0.92 gm/dL, OR = 0.038, p = 0.002). Other factors such as the etiologies of acute abdomen, history of smoking or any other substance use, BMI, intraoperative contamination, and hematologic and biochemical parameters showed no significant association with the development of SSI (p > 0.05). The significantly increased risk of SSI as shown by decreased serum protein levels needs further evaluation as one of the previous studies done by Hennessey et al. [17] found that hypoalbuminemia is an independent risk factor for the development of SSI following gastrointestinal surgery and is associated with deeper SSI and prolonged inpatient stay. This could be attributable to the poor nutritional status of the patients with hypoalbuminemia and thus, poor wound healing.

We assessed the patients for any evidence of SSI in patients on the POD 4, POD 7, POD 14, and POD 30 by regular follow-up. We found out that the use of NPD had significantly reduced the incidence of SSI in the case group as compared to conventional postoperative dressings in the control group on the POD 7 but not on the POD 4 or POD 14.

Our results suggested that NPD was better in preventing SSI as compared to the conventional dressing in the postoperative first week. We ascribe our findings to the fact that NPD, when used for the first four days, continuously drains tissue fluid, thus decreasing tissue bacterial levels. This effect continues to persist over the next few days. Also, as reported in the study by Gundel et al. [18], SSI occurs most commonly between the fourth to 10th days with a median of nine days irrespective of the dressing type. This could be one of the reasons for a high incidence of SSI in both groups on the seventh day and since NPD was effective in reducing the SSI, the comparison showed a statistical difference in the occurrence of SSI on the seventh day.

Thereafter, on the POD 14, the SSI occurrence was less in both groups with no significant difference between them. The changing trend of occurrence of SSI from being significant on the seventh day to insignificant on the 14th day suggests that NPD may be better in preventing SSI in the initial days, but with the passage of time, its effect of draining tissue fluid may not persist for a longer duration. Secondly, since the NPD was used only for four days, which was followed by conventional dressing thereafter, the additive effect of negative pressure was not utilized in the second week leading to similar rates of SSI in both groups on the 14th day. Thirdly, it could be attributable to temporary, but incomplete, dead space closure and fluid evacuation during NPD use.

In our study, the overall occurrence of SSI during the 30 days of follow-up was significantly less with the use of NPD as compared to normal dressing (30% vs. 70%, p < 0.05). Our study results demonstrated that prophylactic use of NPDs is associated with a significant reduction in SSI rates in laparotomy wounds done for peritonitis up to 30 days postoperatively.

Our findings correlate with the study by O'Leary et al. [10], where at 30 days postoperatively, there was a significantly higher incidence of SSI in the control group as was seen in our study (8.3% in the case group vs. 32.0% in the control group, p < 0.05). The latest meta-analysis published in November 2018 by Sahebally et al. [19] also demonstrated that the use of NPWT in closed laparotomy incisions for general and colorectal surgery is associated with a reduced incidence of SSI, compared with conventional dressings.

Although our study shows a clear benefit of NPD over normal dressing, the incidence of SSI as seen in our study at various time intervals was more than in other studies. This can be explained as our study was exclusively done on emergency patients with contaminated and dirty wounds as compared to other studies which included a significant number of patients undergoing elective surgeries.

The use of NPD on surgical wounds is still controversial because, in the study by Murphy et al. [11], the 30-day incidence of SSI was not different between conventional dressing (34%) and NPWT (32%) (p = 0.68). Even Shen et al. [14] found an overall SSI of 16% with no significant difference between the two groups. Indeed, Shen et al. contradicted an earlier retrospective review from their own group published in 2013 which suggested NPWT was associated with lower rates of SSI [20].

Some of the discrepancies within the literature may be due to the heterogeneity of operations included in the studies or small sample sizes, different NPWT devices with different pressure settings (range, -75 to -125 mm Hg), and dissimilar duration of application (four to seven days) as used across previous studies. The use of perioperative antibiotic therapy also differed among studies, with some studies continuing therapy postoperatively when required and others only administering a prophylactic dose at induction. Inclusion and exclusion criteria also varied among studies, with some incorporating contaminated and dirty wounds and others excluding these wound categories. Perhaps results also differed because of variable patient populations exposed variably to intestinal flora during different procedures (colorectal resection vs. pancreaticoduodenectomy).

Our study did not show significant differences in the incidence of abdominal wound dehiscence, duration of hospital stay, and VSS. Similar results were also seen in previous similar studies, studying the effect of NPD [10,11,19].

Due to the non-availability of established guidelines for preventing SSIs, there is a need to come up with more reliable effective techniques. In the literature, the prophylactic use of NPDs is still controversial. In our study, we found a significant reduction in the incidence of SSI on the seventh day and the overall incidence of SSI within 30 days postoperatively in the patients receiving NPDs as compared to the control group. There was less incidence of SSI on the fourth and 14th day as well as an incidence of rectus dehiscence but not significant statically. Therefore, taking all factors into account, a further study with detailed subgroup analysis should be done.

Although our study holds notable strengths, including a robust sample size and a randomized controlled design, and is one of the first to focus on emergency surgery patients. However, there are several limitations to consider. While the sample size is relatively good, focusing on patients with a single type of disease might have provided clearer insights into SSI association factors. Additionally, since most previous studies have concentrated on elective surgery patients, direct comparisons with our findings are limited. Lastly, the study did not include a formal cost-benefit analysis of NPD.

## Conclusions

NPD demonstrates a clear advantage over conventional dressing in reducing the incidence of SSIs, particularly evident on the POD 7 with a negative pressure of -75 mmHg. Prophylactic use of NPD can be beneficial in decreasing morbidity, shortening the duration of hospital stay, and achieving better surgical and cosmetic outcomes by mitigating SSI risks in emergency laparotomy cases. Further research is warranted to assess the long-term benefits and cost-effectiveness of this method to better understand its broader applicability.
